# Organic trace elements enhance growth performance, antioxidant capacity, and gut microbiota in finishing pigs

**DOI:** 10.3389/fvets.2024.1517976

**Published:** 2024-12-23

**Authors:** Weiwei Xu, Miao Zhou, Zhikang Yang, Mengli Zheng, Qinghua Chen

**Affiliations:** ^1^College of Animal Science and Technology, Hunan Agricultural University, Changsha, China; ^2^Key Laboratory of Livestock and Poultry Resources (Pig) Evaluation and Utilization, Ministry of Agriculture and Rural Affairs, Shanghai, China; ^3^Engineering Research Center for Feed Safety and Efficient Utilization of Ministry of Education, Changsha, China

**Keywords:** trace minerals, antioxidant capacity, mineral metabolism, mineral element pollution, gut microbiota

## Abstract

Excessive inorganic trace elements are added to livestock and poultry feed to meet the needs of animals, accompanied by frequent occurrence of excretion and gastrointestinal stress. Replacing inorganic trace elements with organic trace elements provides a promising solution to alleviate these problems. Therefore, this study aimed to assess the impact of replacing all inorganic trace elements (ITMs) in feed on the growth performance, meat quality, serum parameters, trace element metabolism, and gut microbiota of finishing pigs. A total of 128 Duroc × Landrace × Yorkshire growing-finishing pigs (33.88 ± 0.62 kg) were assigned to four treatments in a randomized complete block design. Each treatment was divided into four replicates, each containing eight pigs. The control group received a basal diet containing 100% inorganic trace elements, while the experimental groups were provided with diets where all inorganic trace elements were substituted with 30, 50, and 70% organic trace elements. The experiment spanned 56 days. Results indicated that replacing all ITMs with 30, 50, and 70% OTMs demonstrated no adverse effects on average daily feed intake, average daily gain, feed conversion ratio, eye muscle area, backfat thickness, and relative organ weight of finishing pigs compared to the control group. Furthermore, the replacement led to increased serum immunoglobulin A concentration and Cu-SOD enzyme activity, and decreased serum MDA level, and GSH-Px activity in the liver. Notably, 50 and 70% OTMs increased serum Mn-SOD activity, and 70% OTMs increased serum T-AOC content. Moreover, it significantly decreased the excretion of trace elements in feces without compromising their deposition in the muscle. Additionally, replacing 100% ITMs with 30% OTMs resulted in an improved Shannon index of colonic microbiota in finishing pigs. In conclusion, replacing 100% inorganic trace elements with 30, 50, and 70% organic trace elements exhibited no detrimental effects on the performance of finishing pigs. In conclusion, replacing 100% inorganic trace elements with 70% organic trace elements had certain potential to improve the production performance of finishing pigs. This replacement strategy can enhance meat quality, boost antioxidant capacity, reduce trace element excretion, facilitate trace element absorption and deposition, and enhance gut microbiota homeostasis.

## 1 Introduction

Trace mineral elements are crucial for ensuring the healthy growth, productivity, and reproductive capacity of animals. Serving as cofactors, they play a vital role in synthesizing essential enzymes and proteins within the animal organism (1). These elements are currently categorized into two groups based on their sources: organic and inorganic (2). Inorganic trace elements are predominantly found in the form of inorganic salts and are extensively utilized in modern pig farming due to their cost-effectiveness. Nevertheless, their limitations have become increasingly apparent. Inorganic trace elements are susceptible to dissociation and can form complexes with substances like tannins, phytates, oxalates, and silicates, thereby hindering optimal absorption rates and bioavailability (3). Consequently, the excessive amounts added during production surpass the recommended levels outlined in feeding guidelines, resulting in a significant excretion of metal elements in fecal matter, leading to severe environmental and soil contamination (4). Research indicates that pig manure in China contributes to around 30% of the total waste emissions in the livestock sector, with heavy metal discharge from pig farming making up 71.52% of the overall heavy metal emissions from livestock and poultry manure, the highest share among all types of livestock waste (5, 6). Hence, there is an urgent need to develop strategies to minimize the release of trace minerals from pigs.

Enhancing the absorption efficiency of trace elements, while meeting the nutritional demands of animals, is a crucial strategy to reduce trace mineral emissions. Initially denoting organic acids like zinc gluconate and ferrous fumarate, the definition of organic trace elements has broadened to encompass metal amino acid chelates, metal proteinates, and polysaccharide complexes (7, 8). Extensive research underscores the superior stability of organic trace elements in the animal gut, their reduced susceptibility to degradation, and fewer antagonistic interactions with other compounds. This translates to heightened bioavailability within the animal, ensuring that even modest amounts of trace minerals fulfill the animal’s nutritional requirements. Studies indicate that substituting inorganic trace minerals with lower doses of organic trace minerals has no adverse effects on key economic parameters, including production and egg-laying performance, in sows, piglets, broilers, and laying hens (2, 3, 9, 10). Simultaneously, this substitution increased the absorption rate of trace elements and significantly reduced the excretion of mineral micronutrients in manure, thereby contributing to environmental protection (11). In a prior investigation, we observed that replacing all inorganic trace elements in the diet of weaned piglets with 30 to 50% organic trace elements did not impede growth performance and reduced trace element excretion (12). However, another study demonstrated that replacing inorganic trace elements with glycine- or methionine-chelated copper and zinc significantly improved the growth performance of piglets, while also enhancing the utilization of copper and zinc in the feed (13). Nevertheless, there is a paucity of discourse on the impact of substituting inorganic trace minerals with organic trace minerals on muscle trace mineral deposition and gut microbiota in fattening pigs. Additionally, existing literature predominantly focuses on single organic trace elements like zinc methionine, zinc glycine, and copper methionine, with limited evaluation of multiple complex organic compounds (14).

Hence, this study aimed to examine the impact of substituting 100% inorganic trace elements in pig feed with compound organic trace elements at levels of 30, 50, and 70% on growth performance, carcass traits, meat quality, serum parameters, trace element metabolism, and gut microbiota in growing and finishing pigs. This investigation sought to assess the feasibility of replacing inorganic trace elements in feed with reduced organic trace elements.

## 2 Materials and methods

### 2.1 Animal management, diets, and experimental design

A total of 128 growing-finishing pigs (Duroc × Landrace × Yorkshire) with an initial average body weight of 33.88 ± 0.62 kg were assigned to four treatments in a randomized complete block design based on body weight. Each treatment was divided into four replicates, each containing eight pigs. The basal diet (Table 1) was formulated to meet NRC (2012) standards and supplemented with trace minerals excluding Fe, Cu, Zn, Se, I, and Mn. The control group diet contained 100% inorganic trace minerals (sulfates) commonly used in the Chinese pig industry with additional Fe, Cu, Zn, Se, I, and Mn at 80, 6, 100, 0.3, 0.3, and 40 mg/kg, respectively. Organically complexed Fe, Cu, Zn, and Mn were added to the basal diet at levels of 30, 45, and 60% (30% OTMs, 50% OTMs, and 70% OTMs) compared to the control group using MINEXO™ (Table 2). The molecular weight of copper, iron, manganese, and zinc is <500 Da. Furthermore, all amino acids are from high quality plant proteolysis and contains 18 amino acids. The measured values of copper, iron, manganese, and zinc included in the test diet are shown in Table 3. The study lasted 56 days during which feed consumption was monitored weekly, and body weight was measured at the end to calculate average daily gain (ADG), average daily feed intake (ADFI), and feed to gain ratio (F/G). Pigs had *ad libitum* access to feed and water.

**Table 1:** Composition and nutrient levels of experimental diets (air-dry basis).

Ingredients	Content, %	Nutrient levels^b^	Content
Corn	56.00	Digestive energy, kcal/kg	3190.98
Soybean meal (46%)	18.14	Crude protein, %	15.47
Rice bran meal	8.40	Crude fiber, %	3.99
Brown	6.00	Crude fat, %	2.73
Paddy	8.00	Dry matter, %	85.15
Limestone	1.00	Ca, %	0.54
CaHPO_4_	0.30	TP, %	0.54
NaCl	0.40	Available phosphorus, %	0.17
Choline chloride	0.10	SID Lys, %	1.23
L-Lysine hydrochloride	0.50	SIDMet + Cys, %	0.55
DL-Methionine	0.01	SID Thr, %	0.55
Threonine	0.05	SID Met, %	0.27
Premix^a^	1.10	SID Lys, %	1.23
Total	100		

^aThe premix provided the following per kg of diets: VA 12400 IU, VD3 2,800 IU, VE 30 IU, VK 5 mg, VB12 40 μg, VB1 3 mg, VB2 10 mg, nicotinic acid 40 mg, D-pantothenic acid 15 mg, folic acid 1 mg, VB6 8 mg, biotin 0.08 mg, CaI2O6 0.92 mg, Na2SeO3 0.77 mg. bNutrient levels were determined, except that digestible energy, SID Lys, SID Met, SID Thr, and SID Trp were calculated. The crude protein, crude fiber, crude fat, neutral detergent fiber, acid detergent fiber, moisture, ash, Ca, and TP were all determined according to the China National Standard, and the document numbers are GB/T 6432–2018, GB/T 6434–2006, GB/T 6433–2006, GB/T 20806–2006, NY/T 1459–2007, GB/T 6435–2014, GB/T 6438–2007, GB/T6436-2002, and GB/T 6437–2018, respectively.^

**Table 2:** Experiment design.

Items	Forms	100% ITMs	OTMs		
			30%	50%	70%
Cu, mg/kg	Amino acid-chelated		1.8	3.0	4.2
	Sulfates	6			
Fe, mg/kg	Amino acid-chelated		2.4	40	56
	Sulfates	80			
Mn, mg/kg	Amino acid-chelated		12	20	28
	Sulfates	40			
Zn, mg/kg	Amino acid-chelated		30	50	70
	Sulfates	100			

ITMs, inorganic trace minerals; OTMs, organic trace minerals.

**Table 3:** Measured values of trace minerals in experimental diets (air-dry basis).

Items	100% ITMs	OTMs		
		30%	50%	70%
Cu, mg/kg	6.11	2.04	3.21	4.43
Fe, mg/kg	81.76	25.91	42.71	59.83
Mn, mg/kg	40.91	13.83	22.93	28.40
Zn, mg/kg	106.51	32.56	53.89	75.49

ITMs, inorganic trace minerals; OTMs, organic trace minerals.

### 2.2 Sample collection

Fresh fecal samples were collected from each replicate consecutively for 3 days before the end of the trail and stored at −20°C. The collected feces were mixed, dried, and ground. Using the quartering method, 50 g of dry fecal samples were obtained to assess the trace element content. Post-experiment, all pigs were weighed following a 12-h fast. One pig closest to the average body weight in each replicate was selected for slaughter and sampling. Blood samples were collected from anterior vena cava puncture without anticoagulants, centrifuged at 3000 × g for 10 min at room temperature, and then immediately stored at −80°C for serum biochemical parameters analysis. Following stunning, bleeding, dehairing, evisceration, and midline splitting, the hot carcass weight and intestinal weight were measured. Backfat thickness on the left side of the carcass was recorded at multiple regions, and the mean value was noted. Approximately a 15 cm segment of the intestine was ligated near the proximal colon, followed by a precise incision made at the center of the colon using a sterile scalpel. Subsequently, the colonic contents were collected in a 2 mL sterile centrifuge tube, rapidly frozen in liquid nitrogen, and stored at −80°C. Moreover, a muscle sample approximately 10 cm thick was taken from the longissimus muscle between the fifth and sixth ribs on the left side for meat quality evaluation.

### 2.3 Growth performance

Pigs were weighed on day 1 and day 56 of the fattening period, with daily feed intake being recorded. The gathered data were utilized to determine the average daily feed intake (ADFI), average daily gain (ADG), and feed-to-gain ratio (F/G) according to the following formula:

ADFI = total feed intake/ (test days × test number);

ADG = total weight gain/ (number of test days × number of tests);

F/G = ADFI/ ADG.

### 2.4 Organ index

After slaughtering the growing-finishing pigs, the heart, liver, spleen, intestines, and kidneys were excised. Surface tissue fluid was then dried using absorbent paper and weighed. The organ weight percentage was calculated using the formula: organ index (g/kg) = organ weight/body weight.

### 2.5 Meat quality analysis

Meat color was assessed at 45 min and 24 h postmortem using a portable colorimeter (CR-410, Minolta, Chiyoda, Japan) under a D-65 light source. pH values were also measured at these time points postmortem with a portable pH meter (testo-205, Testo, Lenzkirch, Germany). A muscle sample weighing approximately 120 g was steamed over boiling water for 30 min, then cooled on a hook for another 30 min, dried with filter paper, and reweighed (15). Cooking loss was determined by calculating the weight change percentage. Shear force values were assessed using a Warner-Bratzler shear force device (TA.XT Plus, Stable Micro Systems, Godalming, UK) based on previous studies (16).

### 2.6 Serum biochemical parameter analysis

Serum levels of alanine aminotransferase (ALT), aspartate aminotransferase (AST), alkaline phosphatase (ALP), lactate dehydrogenase (LDH), total bile acid (TBA), total protein (TP), albumin (ALB), globulin (GLB), glucose (GLU), total cholesterol (TC), triglycerides (TG), high-density lipoprotein cholesterol (HDL-C), and low-density lipoprotein cholesterol (LDL-C) were quantified using an automated biochemical analyzer and the respective reagents (KHB 450, Shanghai Kehua Bioengineering Co., Ltd., Shanghai, China).

### 2.7 Immunoglobulin analysis

Serum levels of IgA, IgG, and IgM were determined using ELISA kits following the manufacturer’s instructions (Jiangsu Meimian Industrial Co., Ltd., Yancheng, China). All detection procedures were carried out in accordance with the manufacturer’s instructions for each respective kit.

### 2.8 Antioxidant capacity analysis

Malondialdehyde (MDA, cat: A003-2), total antioxidant capacity (T-AOC, cat: A015-2-1), total superoxide dismutase (T-SOD, cat: A001-2), copper-zinc superoxide dismutase (Cu/Zn-SOD, cat: A001-2), manganese superoxide dismutase (Mn-SOD, cat: A001-2), and glutathione peroxidase (GSH-px, cat: A005) levels were analyzed using commercial kits obtained from Nanjing Jiancheng Bioengineering Institute (NJJCBIO, Nanjing, China). Liver tissue samples (0.1–0.2 g) were excised and rinsed in cold physiological saline to remove residual blood. The tissues were blotted dry using filter paper and accurately weighed. Each sample was placed into a 5 mL homogenizer tube, and 0.86% physiological saline was added at a weight-to-volume ratio of 1:9. Using ophthalmic scissors, the tissue was finely minced, followed by homogenization to prepare a 10% tissue homogenate. Homogenization was performed in an ice-water bath using a tissue grinder at 15,000 rpm for 5 cycles, with each cycle consisting of 10 s of homogenization followed by a 30-s interval. All procedures were conducted under low-temperature conditions to preserve sample integrity. Liver tissue samples were homogenized in physiological saline, and the resulting supernatant was collected post-centrifugation for subsequent analysis. The final results were adjusted based on the total tissue protein concentration.

### 2.9 Trace mineral element analysis

Approximately 15.0 g of *longissimus dorsi* muscle was freeze-dried and ground, and fecal samples were dried in an oven and ground. A 0.20 g portion of the ground sample was placed in a microwave digestion vessel, to which 10 mL of concentrated nitric acid and 1 mL of hydrogen peroxide were added. The sample underwent digestion in a microwave digestion system. Subsequently, 0.5 mL of perchloric acid was introduced to the vessel, and the solution was heated at 180°C for 2 h to eliminate residual acids. The solution was then diluted to 10 mL with 1% nitric acid, and the concentrations of Cu, Fe, Mn, and Zn in the sample were determined using inductively coupled plasma optical emission spectrometry (5,110 ICP-OES, Agilent, US).

### 2.10 Microbial analysis

Microbial diversity in colonic contents was assessed through high-throughput sequencing of bacterial 16S rDNA. The total DNA of intestinal flora in colon contents was extracted using the Cetyltrimethylammonium bromide (CTAB) method. Subsequently, PCR amplification was carried out following electrophoresis detection and quantification. For this purpose, the Primers 341F (5’-CCTACGGGNGGCWGCAG-3′) and 805R (5’-GACTACHVGGGTATCTAATCC-3′) were utilized. The resulting amplified products were purified using AMPure Xbeads and measured with a Qubit. Evaluation of the purified amplified products was conducted using the Agilent 2100 Bioanalyzer and the lumina library quantification kit from Kapa Biosciences. The lumina MiSeq sequencing platform from Shanghai Meiji Biomedical Technology Co., Ltd. facilitated two-end sequencing. Subsequently, the double-end data obtained from sequencing were processed by splitting, concatenating, filtering, and denoising using the DADA2 algorithm to obtain the final Amplicon Sequence Variant (ASV) signature sequence and ASV abundance table. Further analysis, including alpha diversity, species differentiation, and functional prediction, was performed based on the characteristic sequences and abundance tables of ASVs. The significance of alpha diversity was assessed using the Kruskal-Wallis test. Differential intestinal flora were identified through Linear Discriminant Analysis Effect Size (LEfSe) analysis, whereby a significance level of *p* < 0.05 and a Linear Discriminant Analysis (LDA) value >4 were considered as the criteria for differentiation. Additionally, the PICRUSt2 software was utilized for predicting the function of gut microbiota.

### 2.11 Statistical analysis

Data were managed and analyzed using the IBM SPSS Statistics V26.0 software package (IBM Corp., Armonk, NY, USA), with results reported as the mean and pooled standard error. Prior to conducting group comparison analyses, the normality of the data was confirmed through the Shapiro–Wilk test. In cases where the data exhibited a non-normal distribution, statistical analysis involved a one-way ANOVA followed by the Kruskal–Wallis test with multiple FDR corrections. For normally distributed data, a one-way ANOVA analysis followed by LSD test was applied. Significant differences were defined at *p* < 0.05, with a trend toward significance noted at 0.05 ≤ *p* < 0.10.

## 3 Results

### 3.1 Performance, carcass traits, and organ index

The average daily gain, average daily feed intake, feed-to-gain ratio, carcass weight, loin eye area, and backfat thickness of fattening pigs were not influenced by either the level or source of trace element supplementation, as depicted in Table 4 (*p* > 0.10). Moreover, the substitution of 100% ITMs with 30–70% OTMs in Table 5 showed no significant impact on the heart, liver, spleen, kidney, or intestinal indices of fattening pigs (*p* > 0.10).

**Table 4:** Effects of the dietary replacement of inorganic trace minerals with lower levels of organic trace minerals on the growth performance and carcass traits of growing-finishing pigs.

Items	100% ITMs	OTMs			SEM	*p*-value
		30%	50%	70%		
Initial BW, kg	33.94	33.78	33.81	33.81	0.49	0.992
Final BW, kg	79.53	80.73	77.34	80.00	3.96	0.843
ADG, kg/d	0.76	0.75	0.78	0.82	0.05	0.614
ADFI, kg/d	2.01	1.92	1.93	2.04	0.13	0.832
F/G	2.64	2.56	2.49	2.48	0.07	0.168
Carcass weight, kg	27.82	30.26	34.24	35.22	1.25	0.098
Loin eye muscle area, cm^2^	27.82	30.26	35.22	37.00	1.53	0.097
Average back-fat thickness, mm	14.12	13.77	9.44	8.08	1.10	0.070

Values are presented as mean and pooled SEM, *n* = 6. Data in the same row with no or the same letter indicate no significant difference (*P* > 0.05), while with different letters mean significant difference (*P* < 0.05). ITMs, inorganic trace minerals; OTMs, organic trace minerals; BW, body weight; ADG, average daily gain; ADFI, average daily feed intake; F/G, feed-to-gain ratio.

**Table 5:** Effects of dietary replacing inorganic with lower levels of organic trace minerals on the organ index of finishing pigs.

Items	100% ITMs	OTMs			SEM	*P*-value
		30%	50%	70%		
Heart index, g/kg	3.60	3.90	4.00	4.11	0.16	0.748
Liver index, g/kg	17.11	17.61	17.28	16.44	0.64	0.924
Spleen index, g/kg	2.01	2.22	1.87	1.89	0.13	0.781
Kidney index, g/kg	1.47	1.67	1.49	1.69	0.06	0.435
Intestinal index, g/kg	4.42	4.25	4.28	4.80	0.16	0.673

Values are presented as mean and pooled SEM, *n* = 6. Data in the same row with no or the same letter indicate no significant difference (*P* > 0.05), while with different letters mean significant difference (*p* < 0.05). ITMs, inorganic trace minerals; OTMs, organic trace minerals.

### 3.2 Meat quality

As shown in Table 6, the *longissimus dorsi* muscle color, pH at 45 min, shear force, and cooking loss in fattening pigs were not influenced by the source or level of trace element supplementation in the diet (*p* > 0.10). Notwithstanding, the pH at 24 h in the 50% OTMs and 70% OTMs groups showed a significantly higher level compared to the 100% ITMs group (*p* < 0.05).

**Table 6:** Effects of the dietary replacement of inorganic trace minerals with lower levels of organic trace minerals on the meat quality traits of growing-finishing pigs.

Items	100% ITMs	OTMs			SEM	*P*-value
		30%	50%	70%		
Meat color						
L*_45min_	49.67	48.08	47.91	50.95	0.49	0.061
a*_45min_	13.54	14.22	13.78	13.47	0.20	0.606
b*_45min_	6.34	6.12	6.18	6.94	0.19	0.462
L*_24h_	52.38	51.44	55.17	53.07	0.70	0.309
a*_24h_	13.68	13.49	13.49	13.34	0.25	0.980
b*_24h_	4.96	5.84	6.05	5.90	0.29	0.608
pH value						
pH_45min_	6.65	6.66	6.76	7.07	0.10	0.402
pH_24h_	5.58^b^	5.63^b^	6.31^a^	6.59^a^	0.14	0.002
Drip loss						
Shear force, N	78.36	73.97	67.59	51.47	5.09	0.278
Moisture, %	0.71	0.72	0.72	0.80	0.02	0.275

Values are presented as mean and pooled SEM, *n* = 6. Data in the same row with no or the same letter indicate no significant difference (*P* > 0.05), while with different letters mean a significant difference (*P* < 0.05). ITMs, inorganic trace minerals; OTMs, organic trace minerals; L∗, lightness; a∗, redness; b∗, yellowness.

### 3.3 Serum biochemical parameters and immunoglobulin contents

Tables 7, 8 illustrates that substituting 100% ITMs with reduced OTMs in dietary supplementation had no significant impact on the serum levels of ALT, AST, LDH, TBA, TP, ALB, GLB, A/G, BUN, TC, HDL-C, LDL-C, IgG, and IgM (*p* > 0.10). Furthermore, IgA and TG levels were significantly higher in the 30, 50, and 70% OTM groups compared to the 100% ITM group (*p* < 0.05). Notably, 70% OTMs exhibited a marked reduction in serum ALP activity (*p* < 0.05).

**Table 7:** Effects of the dietary replacement of inorganic trace minerals with lower levels of organic trace minerals on the serum biochemical parameters of growing-finishing pigs.

Items	100% ITMs	OTMs			SEM	*P*-value
		30%	50%	70%		
ALT (999U/mg prot)	58.29	55.20	46.33	62.82	2.91	0.242
AST (999U/mg prot)	60.74	56.40	53.00	46.27	2.38	0.168
ALP (999U/mg prot)	151.70^a^	154.99^a^	105.30^b^	156.80^a^	6.85	0.000
LDH (999U/mg prot)	1009.00	1117.00	1127.33	1227.33	71.12	0.810
TBA (999U/mg prot)	36.08	33.85	45.34	43.15	7.97	0.964
TP (999U/mg prot)	79.32	74.90	76.32	84.35	2.27	0.533
ALB (999U/mg prot)	44.41	44.41	44.08	49.21	1.17	0.387
GLB (999U/mg prot)	35.00	30.67	32.33	35.00	1.75	0.824
GLU (999U/mg prot)	5.38^ab^	3.46^c^	4.43^b^	6.13^a^	0.36	0.017
TC (999U/mg prot)	2.74	2.91	3.02	3.00	0.08	0.611
TG (999U/mg prot)	0.73^b^	4.38^a^	3.43^a^	5.23^a^	0.59	0.010
HDL-C (999U/mg prot)	0.83	0.93	0.76	0.85	0.02	0.078
LDL-C (999U/mg prot)	1.35	1.48	1.92	1.61	0.09	0.157

Values are presented as mean and pooled SEM, *n* = 6. Data in the same row with no or the same letter indicate no significant difference (*P* > 0.05), while data with different letters indicate a significant difference (*P* < 0.05). ITMs, inorganic trace minerals; OTMs, organic trace minerals; ALT, alanine aminotransferase; AST, aspartate transaminase; ALP, alkaline phosphatase; LDH, Lactate dehydrogenase; TBA, Total serum bile acids; TP, total protein; ALB, albumin; GLB, globin; GLU, glucose; TC, total cholesterol; TG, triglyceride; HDL-C, high-density lipoprotein cholesterol; LDL-C, low-density lipoprotein cholesterol.

**Table 8:** Effect of low levels of organic trace elements on serum immune indexes of growing fertile pigs.

Items	100% ITMs	OTMs			SEM	*P*-value
		30%	50%	70%		
IgA (μg/mL)	12.73^b^	15.79^a^	14.69^a^	15.29^a^	0.42	0.020
IgG (μg/mL)	20.24	19.97	17.91	20.16	0.46	0.235
IgM (μg/mL)	1.18	1.12	1.08	1.18	0.02	0.263

Values are presented as mean and pooled SEM, *n* = 6. Data in the same row with no or the same letter indicate no significant difference (*P* > 0.05), while with different letters mean a significant difference (*P* < 0.05). ITMs, inorganic trace minerals; OTMs, organic trace minerals.

### 3.4 Antioxidant capacity

As shown in Table 9, the serum MDA levels and CuZn-SOD activity were significantly lower in the 30, 50, and 70% OTMs groups compared to the control group (*p* < 0.05). Conversely, the Mn-SOD activity and T-AOC levels in the 70% OTMs group were significantly higher than those in the control group (*p* < 0.05). No significant differences were found in serum GSH-px and T-SOD activities among the groups (*p* > 0.10). Furthermore, the liver GSH-px activity was significantly higher in all experimental groups than in the control group (*p* < 0.05). Additionally, the liver CuZn-SOD and T-SOD activities were significantly higher in the 50 and 70% OTMs groups compared to the control group (*p* < 0.05). There were no significant differences in liver T-AOC content, MDA content, or Mn-SOD activity among the groups (*p* > 0.10).

**Table 9:** Effects of the dietary replacement of inorganic trace minerals with lower levels of organic trace minerals on the antioxidant capacity of growing-finishing pigs.

Items	100% ITMs	OTMs			SEM	*P*-value
		30%	50%	70%		
Serum						
T-AOC (mM/L)	0.58^b^	0.68^ab^	0.67^ab^	0.73^a^	0.02	0.040
MDA (nM/mL)	1.65^a^	1.46^b^	1.44^b^	1.36^b^	0.04	0.029
GSH-px (IU/L)	509.53	532.40	628.97	684.88	32.31	0.177
CuZn-SOD (IU/mL)	89.96^b^	109.47^a^	116.64^a^	108.47^a^	3.71	0.035
Mn-SOD (IU/mL)	48.67^a^	35.46^ab^	22.43^bc^	16.21^c^	4.23	0.004
T-SOD (IU/mL)	124.67	138.63	139.08	144.93	3.42	0.191
Liver						
T-AOC (nM/g prot)	0.06	0.06	0.06	0.07	0.003	0.664
MDA (nM/mg prot)	0.13	0.14	0.11	0.09	0.002	0.318
GSH-Px (IU/mg prot)	31.10^c^	35.06^b^	37.94^b^	41.56^a^	0.59	0.001
CuZn-SOD (IU/mg prot)	368.73^c^	375.66^bc^	390.57^ab^	398.21^a^	4.43	0.037
Mn-SOD (IU/mg prot)	78.19	82.33	81.65	83.29	0.84	0.136
T-SOD (IU/mg prot)	446.92^c^	457.99^bc^	472.22^ab^	481.50^a^	4.76	0.017

Values are presented as mean and pooled SEM, *n* = 6. Data in the same row with no or the same letter indicate no significant difference (*P* > 0.05), while data with different letters indicate a significant difference (*P* < 0.05). ITMs, inorganic trace minerals; OTMs, organic trace minerals; T-AOC, total antioxidant capacity; MDA, malondialdehyde; GSH-px, glutathione peroxidase; prot, protein; CuZn-SOD, copper-zinc superoxide dismutase; Mn-SOD, manganese superoxide dismutase; T-SOD, total superoxide dismutase.

### 3.5 Contents of trace elements in feces and muscles

Table 10 illustrates that fecal trace element concentrations were notably lower in the 30, 50, and 70% OTMs groups compared to the 100% ITMs group (*p* < 0.05). Conversely, no significant variations were observed in the trace element content of the *longissimus dorsi* muscle across the groups. Noteworthy, the Mn and Zn content in the muscle exhibited an upward trend with increased OTMs supplementation (0.05 < *p* < 0.10).

**Table 10:** Effect of lower levels of inorganic trace minerals on fecal trace element excretion and muscle trace element deposition in growing and fattening pigs.

Items	100% ITMs	OTMs			SEM	*P*-value
		30%	50%	70%		
Fecal excretion						
Cu, mg/kg	54.85^a^	35.22^c^	37.63^c^	40.30^b^	1.64	<0.001
Fe, mg/kg	877.67^a^	430.48^d^	464.59^c^	561.95^b^	37.18	<0.001
Mn, mg/kg	328.95^a^	167.78^d^	189.32^c^	226.78^b^	13.12	<0.001
Zn, mg/kg	981.68^a^	461.72^d^	566.28^c^	622.76^b^	40.89	<0.001
Muscle						
Cu, mg/kg	0.51	0.46	0.49	0.55	0.02	0.278
Fe, mg/kg	5.10	5.14	5.33	5.73	0.18	0.558
Mn, mg/kg	0.52	0.37	0.42	0.52	0.02	0.087
Zn, mg/kg	8.04	7.71	8.53	8.92	0.18	0.049

Values are presented as mean and pooled SEM, *n* = 6. Data in the same row with no or the same letter indicate no significant difference (*P* > 0.05), while data with different letters indicate a significant difference (*P* < 0.05). ITMs, inorganic trace minerals; OTMs, organic trace minerals.

### 3.6 Microbial analysis

#### 3.6.1 Dilution curve

**Figure 1:**
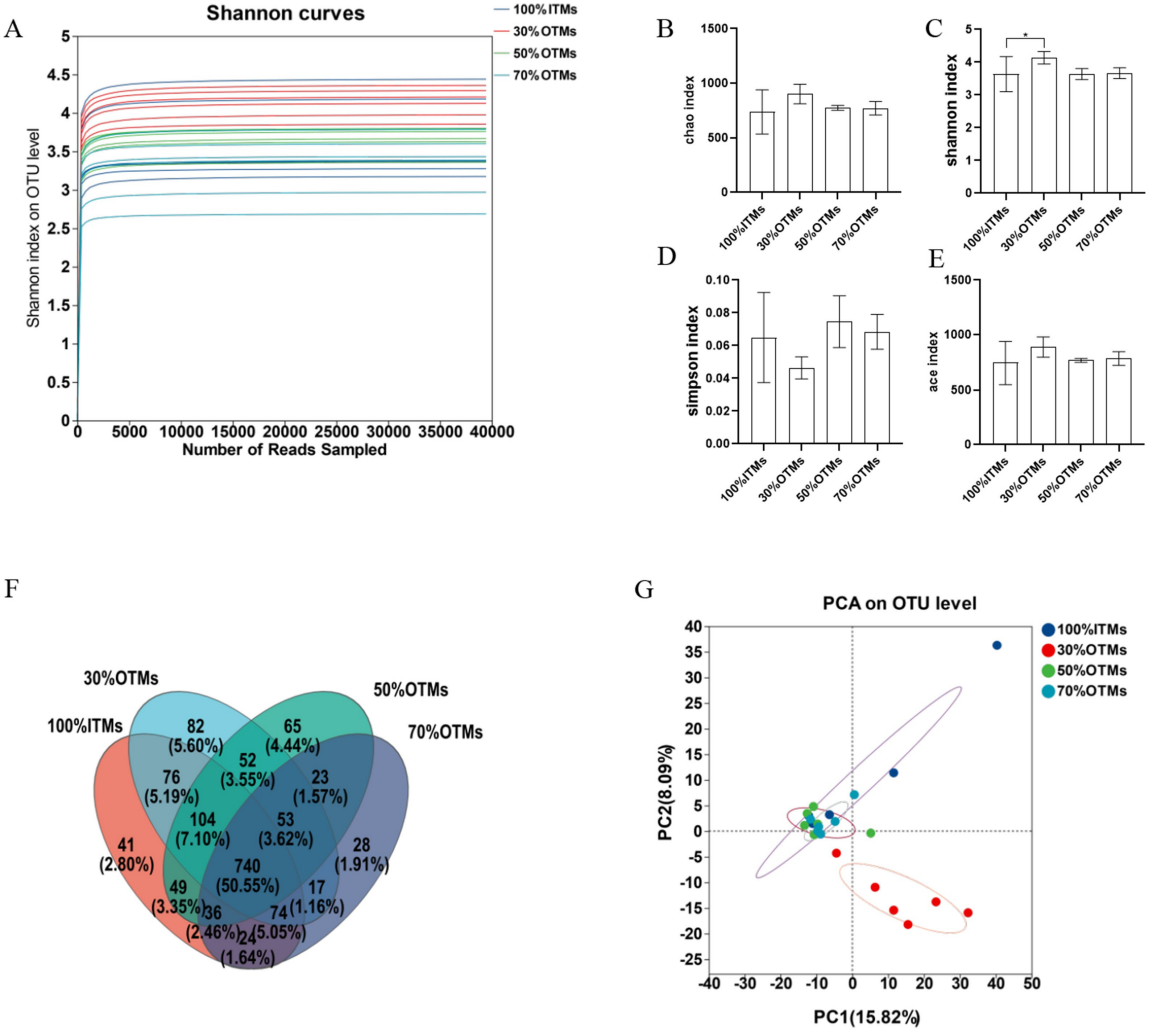
The effects of reducing inorganic trace elements with organic trace elements on the microbial diversity in the colon of finishing pigs. **(A)** Shannon curves, **(B)** Chao index, **(C)** Shannon index, **(D)** Simpson index, **(E)** ace index, **(F)** Wayne figure, **(G)** Principal component analysis (PCA) plot of bacterial communities.

Figure 1A illustrates that the dilution curve starts to level off once the sample size reaches 5,000, signaling that the microbial communities’ detection in the samples is adequate for microbial flora analysis. Figure 1F reveals a total of 1,464 operational taxonomic units (OTUs) identified across the four groups, with 740 OTUs being common to all groups. The counts of OTUs in the 100% ITMs, 30% OTMs, 50% OTMs, and 70% OTMs groups were 1,144, 1,198, 1,122, and 995, respectively.

#### 3.6.2 Gut microbiota diversity

As shown in Figures 1B–E, there were no significant differences in the Ace index, Simpson index, and Chao index of fecal microbiota between the 30% OTMs, 50% OTMs, and 70% OTMs groups compared to the 100% ITMs group (*p* > 0.05). However, the Shannon index of the fecal microbiota in the 30% OTMs group was significantly higher than that in the 100% ITMs group (*p* < 0.05). When the contribution rate of principal component 1 was 15.82% and that of principal component 2 was 8.09%, the 30% OTMs group was distributed further from the 100% ITMs, 50% OTMs, and 70% OTMs groups, indicating a significant difference in microbial diversity between the 30% OTMs group and the other three groups (Figure 1G).

#### 3.6.3 Gut microbial composition

**Figure 2:**
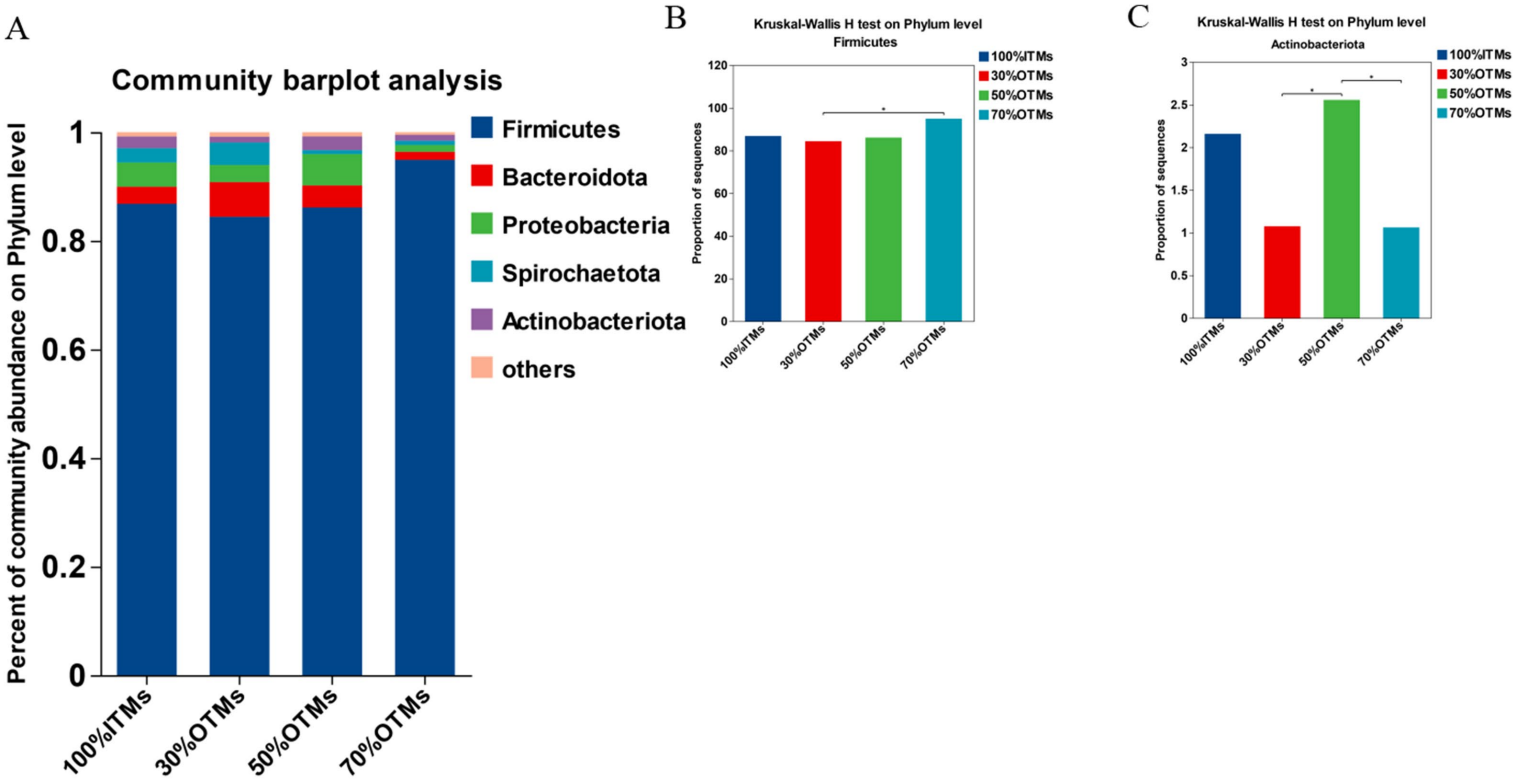
Analysis of phylum-level differences in colonic microbiota of finishing pigs following the replacement of inorganic trace elements with reduced levels of organic trace elements.

As shown in Figure 2A, the top five phylum-level microbial abundances in the 100% ITMs, 30% OTMs, 50% OTMs, and 70% OTMs groups were *Firmicutes*, *Bacteroidota*, *Proteobacteria*, *Spirochaetota*, and *Actinobacteriota*, respectively. According to Figures 2B,C, the abundance of *Firmicutes* was significantly higher in the 70% OTMs group compared to the 30% OTMs group (*p* < 0.05), with no significant differences observed among the other groups (*p* > 0.05). Additionally, the abundance of *Actinobacteriota* was significantly higher in the 50% OTMs group compared to the 30% OTMs and 70% OTMs groups (*p* < 0.05).

**Figure 3:**
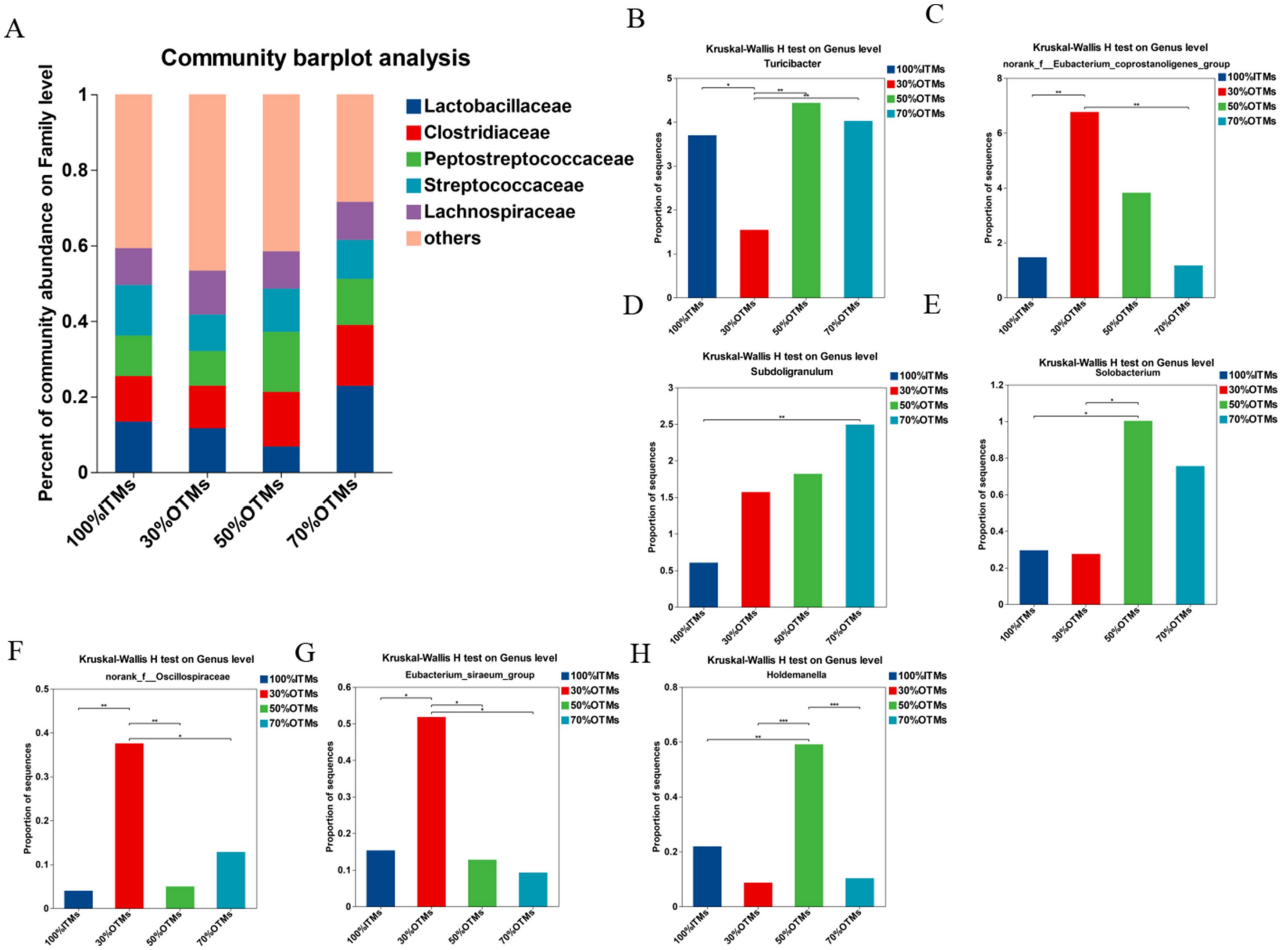
Analysis of family-level differences in colonic microbiota of finishing pigs following the replacement of inorganic trace elements with reduced levels of organic trace elements.

As shown in Figure 3A, at the genus level, the top five microbial compositions in the 100% ITMs group were *Lactobacillus* (13.40%), *Streptococcus* (13.39%), *Clostridium_sensu_stricto_1* (11.94%), *UCG-005* (11.62%), and *Terrisporobacter* (10.70%). In the 30% OTMs group, the top five were *Lactobacillus* (11.63%), *Clostridium_sensu_stricto_1* (10.95%), *Streptococcus* (9.65%), *Terrisporobacter* (9.14%), and *UCG-005* (8.01%). For the 50% OTMs group, the top five were *Terrisporobacter* (15.89%), *Clostridium_sensu_stricto_1* (14.35%), *Streptococcus* (11.42%), *UCG-005* (7.61%), and *Lactobacillus* (6.80%). In the 70% OTMs group, the top five were *Lactobacillus* (22.86%), *Clostridium_sensu_stricto_1* (15.99%), *Terrisporobacter* (12.19%), *Streptococcus* (10.30%), and *UCG-005* (6.98%).

According to Figures 3B–H, compared to the 100% ITMs group, the relative abundance of *Turicibacter* in the 30% OTMs group was significantly lower (*p* < 0.05), while the relative abundances of *Eubacterium_siraeum_group*, *norank_f__Eubacterium_coprostanoligenes_group* and *norank_f__Oscillospiraceae* were significantly higher (*p* < 0.05). The relative abundance of *NK4A214_group* in the 100% ITMs group was significantly higher than in the 50% OTMs and 70% OTMs groups (*p* < 0.05). The relative abundance of *Solobacterium* in the 50% OTMs group was significantly higher than in the 100% ITMs and 30% OTMs groups (*p* < 0.05). Additionally, the relative abundance of *Holdemanella* in the 50% OTMs group and *Eubacterium_siraeum_group* in the 30% OTMs group were significantly higher than in the other groups (*p* < 0.05).

#### 3.6.4 Correlation analysis between gut microbiota and serum antioxidant indicators, serum immune indicators, and fecal trace element content in fattening pigs

**Figure 4:**
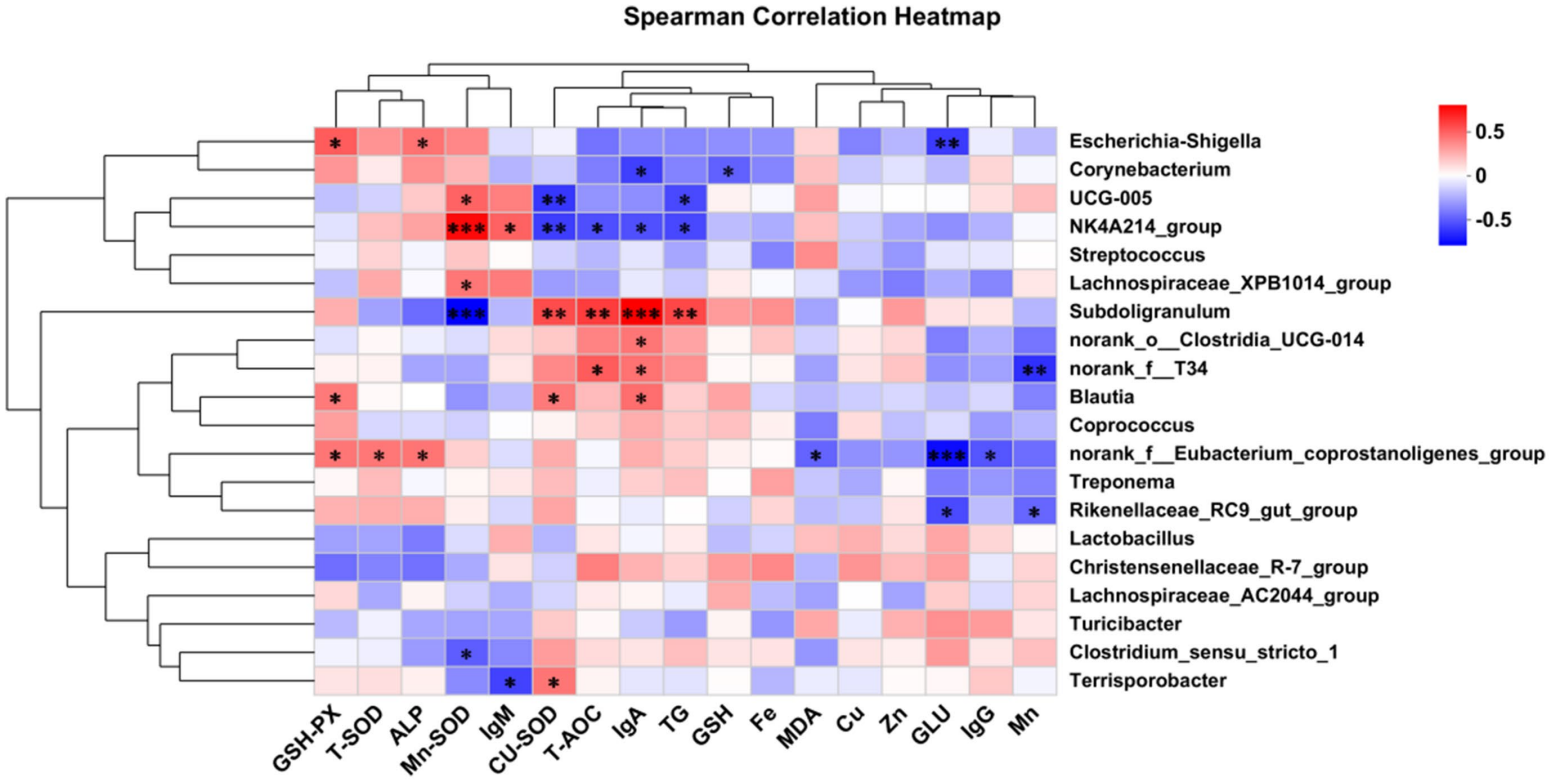
Correlation analysis of microbial species with muscle trace element deposition, immune indicators, serum antioxidant capacity, and serum biochemical parameters. GSH-PX, Glutathione peroxidase; T-SOD, Superoxide dismutase; ALP, Alkaline phosphatase; Mn-SOD, Mn Superoxide dismutase; lgM, immunoglobulin M; Cu-SOD, Cu Superoxide dismutase; T-AOC, Total antioxidant capacity; lgA, immunoglobulin A; TG, Triglyceride; GSH, Glutathione; Fe, Iron content in longissimus dorsi muscle; MDA, Malondialdehyde; Cu, Copper content in *longissimus dorsi* muscle; Zn, Zinc content in *longissimus dorsi* muscle; GLU, Glucose; lgG, immunoglobulin G; Mn, Manganese content in *longissimus dorsi* muscle. Differences were set to *p < 0.05 and **p < 0.01.

Spearman correlation analysis was also conducted to investigate the relationships among serum antioxidant index, immunoglobulin levels, fecal trace element content, and the gut microbiota of the piglets (Figure 4). In the gut microbiota, *Escherichia-Shigella*, *Blautia*, and *norank_f__Eubacterium_coprostanoligenes_group* were significantly positively correlated with GSH-px activity (*p* < 0.05).

There were significant positive correlations between *norank_f__Eubacterium_coprostanoligenes_group* and T-SOD activity (*p* < 0.05). ALP activity was significantly positively correlated with both *Escherichia-Shigella* and *norank_f__Eubacterium_coprostanoligenes_group* (*p* < 0.05). Mn-SOD activity showed significant positive correlations with *UCG-005*, *NK4A214_group*, and *Lachnospiraceae_XPB1014_group* (*p* < 0.05), and significant negative correlations with *Subdoligranulum* and *Clostridium_sensu_stricto_1* (*p* < 0.05). Serum IgM levels were significantly positively correlated with *NK4A214_group* (*p* < 0.05) and significantly negatively correlated with *Terrisporobacter* (*p* < 0.05). CuZn-SOD activity had significant positive correlations with *Subdoligranulum*, *Blautia*, and *Terrisporobacter* (*p* < 0.05), and significant negative correlations with *UCG-005* and *NK4A214_group* (*p* < 0.05). T-AOC content was significantly positively correlated with *Subdoligranulum* and *norank_f__T34* (*p* < 0.05), and significantly negatively correlated with *NK4A214_group* (*p* < 0.05). IgA levels showed significant positive correlations with *Subdoligranulum*, *norank_o__Clostridia_UCG-014*, *norank_f__T34*, and *Blautia* (*p* < 0.05), and significant negative correlations with *Corynebacterium* and *NK4A214_group* (*p* < 0.05). TG content had a significant positive correlation with *Subdoligranulum* (*p* < 0.05), and significant negative correlations with *UCG-005* and *NK4A214_group* (*p* < 0.05). GSH content was significantly negatively correlated with *Corynebacterium* (*p* < 0.05). MDA content showed a significant negative correlation with *norank_f__Eubacterium_coprostanoligenes_group* (*p* < 0.05). GLU content was significantly negatively correlated with *Escherichia-Shigella*, *norank_f__Eubacterium_coprostanoligenes_group*, and *Rikenellaceae_RC9_gut_group* (*p* < 0.05). IgG levels were significantly negatively correlated with *norank_f__Eubacterium_coprostanoligenes_group* (*p* < 0.05). Fecal Mn excretion was significantly negatively correlated with *norank_f__T34* and *Rikenellaceae_RC9_gut_group* (*p* < 0.05).

## 4 Discussion

In contemporary traditional intensive pig farming, trace elements are often added in excess to pig diets to prevent deficiencies, while the naturally occurring trace elements in the feed are frequently disregarded. The NRC (2012) stipulates that for fattening pigs (25–80 kg), the required trace element levels are Cu 3.50 mg/kg, Fe 50.00 mg/kg, Mn 2.00 mg/kg, and Zn 50 mg/kg. In this study, the basal diet in this experiment adhered to the NRC standards for the control group. Moreover, all trace elements in the 70% organic trace element substitution group met the NRC standards, whereas in the 50% substitution group, Mn and Zn levels exceeded the standards, and in the 30% substitution group, Mn levels surpassed the recommended levels. The levels of the other trace elements fell below the NRC standards. Recent research suggests that replacing 50% of the NRC standard inorganic trace elements (Cu, Fe, Mn, Zn) with organic trace elements has no significant impact on the growth performance, slaughter performance, or backfat thickness of fattening pigs (17). Consequently, we conclude that the feed used in this experiment adequately meets the nutritional needs of the test animals.

A significant body of research suggests that organic trace elements can increase the bioavailability of trace elements in livestock and poultry, enhance production performance, meat quality, and immunity, and decrease the excretion of trace elements in feces, thereby mitigating environmental pollution (11). Due to their superior bioavailability and performance in production, organic trace elements are extensively utilized in pigs (18). Prior studies have demonstrated that substituting 30–60% of inorganic trace elements with organic trace elements in the diet of fattening pigs does not adversely affect their production performance (2). Similarly, using a low dose (one-third) of organic trace elements to entirely replace a high dose (100%) of inorganic trace elements does not negatively impact the production performance of weaned piglets (19). Consistent with earlier research, this study observed that replacing 100% of inorganic trace elements with 30% organic trace elements had no detrimental effect on the growth and slaughter performance of fattening pigs. Conversely, reducing the proportion of inorganic trace elements (100%) by substituting them with organic trace elements (30, 50, and 70%) could potentially enhance slaughter performance, increase loin eye area, and reduce backfat thickness in fattening pigs.

The organic trace elements utilized in this study were amino acid chelated trace elements, which are abundant in various amino acids and are more readily absorbed by animals. Furthermore, this experiment indicated that once organic trace elements fulfill the growth requirements of the animals, excessive supplementation does not enhance the production performance of fattening pigs. Replacing inorganic trace elements with organic counterparts in the diet shows promise in enhancing meat quality in livestock and poultry. Previous research implies that organic trace elements might enhance meat quality in fattening pigs through the activation of antioxidant enzymes (20). The inclusion of yeast chromium in the diet can notably decrease lipid peroxidation in fattening pigs, hence improving meat tenderness and antioxidant capacity (21). Moreover, studies have revealed that substituting 12.5% of inorganic trace elements with organic variants can considerably reduce the a* value of breast muscle in white-feather broilers and lower abdominal fat percentage. In alignment with these findings, this study determined that employing 50 and 70% organic trace elements instead of inorganic ones resulted in a significant increase in 24-h muscle pH compared to the 100% organic trace element group. This indicates the potential of substituting inorganic trace elements with organic ones for enhancing meat quality.

Previous research has shown that trace elements from diverse sources do not notably impact serum biochemistry in livestock and poultry; however, they can enhance immune function. For instance, substituting one-third of inorganic trace elements with commercial-level organic alternatives can markedly elevate serum IgG levels in fattening pigs while not significantly altering other serum biochemical parameters (12). Similarly, the replacement of inorganic selenium with organic selenium has been reported to have no noteworthy effect on serum biochemical parameters in laying hens (22). In deviation from earlier studies, this investigation reveals that substituting inorganic trace elements with 30% organic counterparts leads to a significant reduction in serum glucose levels in fattening pigs, whereas replacements of 50 and 70% show no substantial impact. Additionally, all levels of organic trace element substitution (30, 50, and 70%) result in a remarkable increase in serum TG and IgA levels in fattening pigs. TG, linked to lipid metabolism and fat deposition, acts as a vital energy source for animals. The findings observed may be attributed to the utilization of amino acid chelated organic trace elements in this study, which encompass various amino acids absorbed in the small intestine and utilized by the body for lipid metabolism. Moreover, the surge in serum IgA levels with organic trace element supplementation could be associated with the activation of the immune system by these trace elements.

Antioxidant function is a vital system for maintaining the health of livestock and poultry, with Cu, Fe, Mn, and Zn serving as essential components of antioxidant enzymes in animals. Research has demonstrated that substituting 100% of inorganic trace elements with 30% organic counterparts can notably boost serum T-SOD and muscle Mn-SOD enzyme activity in fattening pigs, thereby enhancing their overall antioxidant capacity (2). Prior studies have shown that supplementing the diet with 70% organic trace elements significantly elevates serum glutathione peroxidase activity in laying hens. Moreover, incorporating 40–70% organic trace elements can significantly increase the total antioxidant capacity in the serum of laying hens (23). Similarly, the findings of this study suggest that replacing 100% of inorganic trace elements with 70% organic elements significantly raises serum T-AOC levels and Cu-SOD enzyme activity in fattening pigs while decreasing serum MDA levels. Furthermore, substituting inorganic trace elements with 50 and 70% of organic versions notably enhances liver GSH-Px, Cu-SOD, and T-SOD enzyme activities. Even with a reduction in organic trace element supplementation to 30%, there is still an enhancement in antioxidant capacity in fattening pigs, as indicated by increased serum Cu-SOD and liver GSH-Px enzyme activities.

Amino acid-chelated trace elements exhibit higher absorption rates than inorganic trace elements due to sharing the same absorption pathways as amino acids. This distinct absorption profile reduces competition and antagonistic effects (12). Numerous studies have indicated that substituting inorganic trace elements with low-dose organic variants can diminish mineral excretion in feces and enhance the efficiency of trace element absorption. For instance, replacing 100% of commercial inorganic trace minerals with 1/3 organic trace elements showed no adverse effects on the production performance, yolk mineral retention, or egg quality of laying hens, while notably decreasing trace element excretion in feces (9). Similarly, a study supplementing the diet of yellow-feathered broilers with organic trace elements revealed higher levels of serum Mn and Se, breast muscle Fe, Zn, and Se, as well as heart Se, compared to the inorganic trace element group. Moreover, fecal mineral excretion was reduced in the organic trace element-fed group (24). In line with prior research, the outcomes of this study illustrate that replacing inorganic trace elements with 30–70% organic counterparts considerably decreases fecal trace element content in fattening pigs without compromising trace element deposition in muscle tissue, offering significant environmental benefit. Forthermore, at a 70% replacement rate, organic trace elements tend to enhance trace element deposition in muscle.

Gut microbiota play a critical role in maintaining animal health by supporting gut health through the diversity of metabolic functions, preservation of the intestinal mucosa integrity, and reinforcement of innate immunity as the primary defense against foreign and toxic substances (25, 26). Studies have shown that trace minerals like Cu, Fe, Mn, and Zn from different sources can enhance gut health by adjusting the composition and function of the intestinal microbiota (27). For instance, substituting copper sulfate with 20 mg/kg of copper glycinate notably increased the colonic microbiota abundance in fattening pigs and decreased trace element emissions (28). In our investigation, we utilized 16S rDNA sequencing to assess the *α*-diversity of colonic microbiota in fattening pigs and observed a significant increase in the Shannon index when replacing 100% ITMs with 30% OTMs. Moreover, PCA analysis based on bray_curti illustrated a substantial difference in microbial composition between the 30% OTMs group and the control group. A higher abundance of beneficial gut bacteria is generally beneficial for host health (29). At the phylum level, the 70% OTMs group exhibited the highest *Firmicutes* abundance. As the organic trace minerals’ proportion rose, Firmicutes’ abundance also increased, while *Campylobacterota*’s relative abundance decreased with the rising replacement ratio. *Campylobacterota* are pathogenic bacteria associated with various diseases in humans and animals. At the genus level, the 30% OTMs group demonstrated significantly higher abundances of *norank_f__Oscillospiraceae*, *norank_f__Eubacterium_coprostanoligenes_group*, and *Eubacterium_siraeum_group* compared to the 100% ITMs group, with the pathogenic genus *Terrisporobacter* significantly less prevalent than in the control group. *Norank_f__Oscillospiraceae* and *Eubacterium_siraeum_group* are commonly found in animal intestines and can produce beneficial short-chain fatty acids like butyrate, essential for gut health. Furthermore, the 50% OTMs group displayed notably higher abundances of *Streptococcus* and *Holdemanella* compared to the 100% ITMs group. *Streptococcus* aids in gut lactose digestion and is crucial for maintaining intestinal wall health, while *Holdemanella* is identified as a potential contributor to gut health (30). Previous studies have found a positive correlation between *Holdemanella* and the digestibility of carbohydrates and fibers (31). The 70% OTMs group showed significantly higher relative abundance of *Subdoligranulum* compared to the 100% ITMs group. *Subdoligranulum* plays a role in lactate conversion to butyrate (32). Overall, our results suggest that replacing inorganic trace elements with organic ones at varying ratios can notably increase the abundance of beneficial gut bacteria, suppress harmful bacteria proliferation, and promote gut health in fattening pigs.

## 5 Conclusion

In this trial, we observed that replacing inorganic trace elements with organic alternatives, under the given feeding conditions, had no adverse effects on growth performance or the relative organ weight in fattening pigs and showed potential for enhancing meat quality. Notably, the substitution improved lipid metabolism, immune function, antioxidant levels, trace element emissions reduction, and gut microbiota structure. In conclusion, replacing 100% inorganic trace elements with 70% organic trace elements had certain potential to improve the production performance of finishing pigs. This study highlights the viability of reducing inorganic trace elements with organic counterparts, which bears substantial implications for trace element reduction and environmental conservation. Further extensive research is essential to elucidate the precise mechanisms of efficient organic trace element absorption.

Ethics approval and consent to participate.

## Data Availability

The datasets presented in this study can be found in online repositories. The name of the repository and accession number can be found below: National Center for Biotechnology Information (NCBI) Sequence Read Archive (SRA), https://www.ncbi.nlm.nih.gov/bioproject/1170518, project number is: PRJNA1170518.
